# News Items

**Published:** 1872-03-15

**Authors:** 


### News Items.

HONORS DECLINED.—Profs. Billroth and Brucke, of Vienna, have been offered chairs in the University of Strasbourg. They have, however, decided on remaining in Vienna.

DEATH OF SIR JAMES MURRAY, BART., M.D. —The death is announced of this veteran of our profession at the ripe old age of eighty-three. He has been for many years Inspector of Anatomy for Ireland, and physician to the Lord-Lieutenant. He was born in 1788, was educated at Edinburgh and at Trinity College, Dublin, and received the honor of knighthood in 1863.—*Dublin Med. Press and Circular.*

A CENTENARIAN PHYSICIAN.—Dr. Francis Hay, of Columbus, Ohio, recently celebrated his one hundredth birthday. The doctor is a native of Bavaria, having been born in Wurzburg, December 8, 1771. He was a student at that place, and there acquired his profession. In 1834 he came to America, and practised his profession successfully in various parts of this country, and in 1861 went to Columbus, where he has resided ever since. His wife, to whom he was married in 1802, is now in her ninety-fifth year, is quite nimble and healthy, and bids fair yet to survive a number of years. The doctor himself is wonderfully lithe and strong for one so old. He is frequently seen upon the streets walking with a firm and steady step, and as upright in his carriage as a man in the prime of life. He is perfectly able to help himself, and in the care of his person asks for no assistance from any one.—*Philada. Med. and Surg. Reporter.*

Appointments, Honors, Etc.—Dr. Billod, chief physician to the lunatic asylum of the Seine, at Epinay-sur-Orge, has received from the inhabitants of his commune a gold medal, purchased by subscription, in recognition of his services during the siege of Paris. The Cross of the Legion of Honor has been sent by the French Government to Madame Pochet, of Havre, for services rendered during the war. The honorary membership of the Royal Society of Sciences of Brussels has been conferred on Sir James Paget and Sir William Ferguson. Dr. Tilbury Fox has been made a member of the Leprosy Committee of the Royal College of Physicians. Mr. John Hilton has been elected President of the Pathological Society of London for the year 1872. A subscription is on foot among the profession in Italy to strike a gold medal to be presented to Virchow. M. Barth has been elected President of the Academy of Medicine, of Paris, for the present year. The degree of LL.D. has been conferred by the Faculty of Princeton College on Drs. Abraham Coles, of Newark, and Hugh L. Hodge, of Philadelphia. Dr. Bamberger has been elected to the chair of Medicine in the University of Vienna, to succeed the late Professor Oppolzer. Dr. Tyson has retired from the position of assistant editor of the *Philadelphia Medical Times.* Dr. Thomas W. Evans, of Paris (an American), has been promoted to the rank of Commander in the Legion of Honor.—*New York Med. Times, Feb.*, 1872.

Small-pox in Hamburg.—According to information furnished by the Hamburg Board of Health, there were in that city, from Aug. 19 to Nov. 18, 1871, 5,707 cases of small-pox, with 1,047 deaths, 696 cases remaining under treatment. The following are the statistics as regards vaccination:

| | Vaccinated. | Unvaccinated. |
|---|---|---|
| Recoveries | 2,954 | 1,010 |
| Deaths | 347 | 700 |

A Memorial to Jenner.—It is proposed to erect a memorial window in the old church at Berkeley, Gloucestershire, England, to perpetuate the fame of Jenner, who lived and died in Berkeley. The sum of £500 is needed for the purpose, and the subscription list is headed by the names of three earls.

### NECROLOGIST'S REPORT.

*To the Alumni Association of the Chicago Medical College:*

Your Necrologist has the honor to present biographical sketches, as full as he has been able to make, of Dr. W. H. Peavler and Jas. F. Flemming; also, an unfinished memoir of Dr. Otho Bonser, all deceased alumni of our *alma mater.* The unfinished paper, it is hoped, will be completed by the next annual meeting. A very full biography of Dr. M. N. Rust will also at that time be presented.

The exceeding brevity of these biographies is a matter for regret, but it is unavoidable. The gathering of facts of the personal history of individuals who have never been notorious, by one often at a distance from friends of the deceased, who is obliged to get facts through letters long delayed, and often brief and insufficient, must be slow and at times necessarily unsatisfactory.

Of the eleven alumni so far reported as deceased, it has been impossible to gain any facts regarding the following five: J. M. Kendall, D. J. Alloban, E. B. Rockwell, J. J. Samuels and Geo. Ware Wilson. To supposed friends of some of them, letters of inquiry have been addressed, but so far no replies have been received. Regarding the others, nothing has been elicited as to where to seek for information. Respectfully submitted, N. Bridge, *Necrologist.*

Chicago, March 12, 1872.

---

### W. H. Peavler.—*Class of* 1865.

Dr. W. H. Peavler was born in Pecan, Washington Co., Ind., August 4, 1838. He was the second of a family of twelve children—seven girls and five boys. His father, Gabriel Peavler, a farmer, was born in Sullivan Co., Tenn., January, 1813; his mother was born in Indiana, in 1819.

Young Peavler removed with his parents to Illinois, in September, 1850. He attended a common district school during winters, spending his summers in work on the farm, until he was sixteen years of age, when he entered a higher school at Marshall, Ill. After remaining here six months, he became a clerk in a drug store. A year from this time found him teaching school not far from Melrose, Ill. On the expiration of his term at this place, he took a school near Spring Garden, whither his father's family had removed. He taught this school six months; he taught afterward six months in another school in the vicinity.

In the spring of 1858 he fell in with the current of popular feeling—credulity about the mining wealth of the region of Pike's Peak. In May of that year he joined the caravan that was long and hopeful, and that was directing its way westward. With others he was disappointed in the mission of his pilgrimage to the mountains, and he returned, injured in health, in December following his departure. He very soon began again his occupation of school teacher, remaining in school another half year.

In the spring of 1859 he purchased a horse and wagon, and set himself up in the peddling business, dealing in dry goods, notions, etc., and buying produce of all sorts.

But at this occupation he did not succeed well, and he only followed it one season. He then sold his outfit, and began at once the study of medicine.

This was in the autumn of 1859. He studied about a year, and taught school the winter following; then he resumed his studies.

In the spring of 1862 he volunteered for the army, and enlisted in the 49th Regiment Illinois Volunteers. He was soon detailed to do duty in Mound City General Hospital, as ward master. In this capacity he served about one year; it was, however, over three years before he finally left the service, and when discharged he had attained the rank of sergeant. During his term of service, Peavler obtained leave of absence, as he supposed, for ninety days. He came home, and immediately repaired to Ann Arbor and entered the medical class. He was ordered back to duty, however, at the end of fifty days, evidently much to his disappointment. This was about April 1, 1864.

Not until after his final discharge from the army was he enabled to take up again his medical college study. He came to Chicago then, and entered the Chicago Medical College, and graduated with the class of 1865. His first course of lectures was thus of only two months' duration, while his last was a full term.

His first shingle was hung out in DuQoin, Ill., with an elderly practitioner by the name of Jones. But the partnership did not last long, and Peavler began business for himself.

In fourteen months from this time—namely September 15, 1866—Dr. Peavler was attacked with cholera and died in ten hours. His was the only case occurring in his town during that season.

Dr. H. Wardner, who was surgeon-in-charge of Mound City Hospital at the time Peavler was there, has written a letter in which he says of him: "He was a young man of delicate constitution, but most excellent character and habits. An honest, faithful physician, he fell a prey to the dread scourge of cholera in the summer of 1866, while at his post amid his patients."

Dr. Peavler was never married. He had been a member of the Sons of Temperance for many years; he was also a member of the order of Good Templars, of the Odd Fellows, and of the Masonic fraternity.

Very curiously, two brothers of Dr. P. had hair and eyebrows perfectly white, and one sister had white hair at birth. None of the rest of the family had this peculiarity.

James Francis Flemming.—*Class of* 1865.

Dr. James F. Flemming was born about the year 1843, on North Dearborn, near Kinzie street, Chicago. His parents were both Irish. His father, Wm. Flemming, was by trade a tailor; he had resided in Chicago many years. He was at one time considerably known in connection with local politics. he died about ten years ago; his widow still survives.

Dr. F. was at a very early day sent to a Catholic school, the school since located on the corner of Superior and N. State streets. He continued here until he was eight years of age, when he was put into the College of the same sect as a boarder—a boarding pupil —where he remained until the College was broken up. Then he was sent to St. Louis and put under the instruction of Rev. Father Dijon. At this time he was about ten years of age. He remained under this teacher two years. He then spent a year in the Brothers' school in St. Louis, when he went to the Cape Girardeau College. Here five full years were spent in study, when the lad returned to Chicago. Not long after his return he was employed as a teacher in the College of the Holy Name, remaining about one year.

His parents had, almost from his infancy, intended him for the priesthood. With this object in view his education had been planned, and had been made thorough.

But about this time he conceived a desire to study medicine. After deliberation, he determined to do this, and entered the office of Prof. Byford as a student. All his lectures were attended in the Chicago College, and he graduated in 1865.

He entered the army in 1864, a few days after his twenty-first birthday; he was connected with the army eleven months, and belonged to a regiment of colored troops.

While in the army he was accidentally thrown from his horse, and so severely injured that he could not longer remain in the service. He was sent home, and died on March 31st, two weeks after his arrival; he died in Mercy Hospital.

Dr. F. was the last survivor of a family of four children. Two died in early childhood. A sister, Ellen M., died only a year before his death—namely, on May 24, 1864.

Dr. F. was never married, nor did he intend to. He entertained a strong hope to the last, which he often expressed, of finally entering the clerical profession. With this view he determined not to marry; but his want of experience in the world, and his high idea of what the priesthood ought to be, made him feel it his duty not to enter it until he became older.

---

It is said that a piece of ice of the size of a horse chestnut, put into the rectum every two hours, will speedily relieve retention of urine.

On the First Insensibility from Ether.—Dr. John H. Packard calls attention to the fact that there is a first insensibility produced on the administration of ether, which is followed by a stage of excitement. This first insensibility may be taken advantage of by the surgeon in the performance of slight operations, like opening whitlows, etc., without the trouble to himself and the patient of carrying the process to complete anæsthesia. A little ether is to be poured on a towel, the patient directed to inhale fully, and at the same time raise the hands, and to keep his attention fixed on them. When the hands drop, do the operation promptly.—*Philadelphia Medical Times*, *Feb.* 15, 1872.

Hypodermic Injection of Morphia in Dysentery.—Cases of dysentery cured by hypodermic injections of morphia alone are recorded by Dr. Thomas J. Gallagher, of Pittsburgh, Pa., in the *N. Y. Medical Journal.* The pain and tenesmus are instantly relieved by this method, and the cure is much quicker than by the usual procedure; also, the administration of frequent doses of nauseous drugs obviated. From one to two injections, mostly but one, daily, is all that is required.—*Ibid.*

Treatment of Diabetes.—Dr. G. Moore, of Hastings, (*British Med. Journal,*) lately read a paper on a case of diabetes of three months standing. The patient had previously been rigorously dieted. Under a bread and milk diet (three pints of the latter daily) and the administration of effervescing salines, with iron, the urine became perfectly free from sugar in a fortnight, and the patient speedily gained flesh and strength. Dr. Moore expressed himself strongly in favor of the free use of milk in such cases.

